# Clinical outcomes of doxorubicin-eluting CalliSpheres beads-transarterial chemoembolization for unresectable or recurrent esophageal carcinoma

**DOI:** 10.1186/s12876-021-01816-3

**Published:** 2021-05-21

**Authors:** Yonghua Bi, Xiaonan Shi, Jianzhuang Ren, Mengfei Yi, Xinwei Han, Min Song

**Affiliations:** 1 Department of Interventional Radiology, The First Affiliated Hospital of Zhengzhou University, No.1, East Jian She Road, Zhengzhou, 450052 China; 2 Department of Oncology, The First Affiliated Hospital of Zhengzhou University, Zhengzhou, China

**Keywords:** Esophageal carcinoma, Drug-eluting beads, Transarterial chemoembolization (TACE), CalliSpheres beads, Doxorubicin

## Abstract

**Background:**

The clinical outcomes of drug-eluting beads transarterial chemoembolization (DEB-TACE) with doxorubicin-loaded CalliSpheres beads for patients with unresectable or recurrent esophageal carcinoma have not been reported. The aim of this study is to study the clinical outcomes of DEB-TACE for patients with unresectable or recurrent esophageal carcinoma.

**Methods:**

This retrospective study enrolled 21 patients (15 men; mean age 68.79.7; range 4686 years) with unresectable or recurrent esophageal carcinoma received DEB-TACE between July 2017 and September 2020. Patient characteristic data, imaging findings, complications and DEB-TACE procedure were reviewed. The primary endpoints, disease control rate (DCR) and objective response rate (ORR), were calculated. The secondary endpoints were overall survival rate and progression-free survival (PFS).

**Results:**

Twenty-two sessions of DEB-TACE were performed in 21 patients. The technical success rate was 100%; without sever adverse events or procedure-related deaths. All patients received transarterial chemotherapy infusion with raltitrexed or oxaliplatin. The median follow-up period was 3.6 months (interquartile range, IQR 1.59.4 months). ORR and DCR were 42.9 and 85.7%, 28.6 and 71.4%, 20.0 and 40.0% respectively at 1-, 3-, and 6-months after DEB-TACE. The median PFS was 6.0 months, and the 3-, 6- and 12-month PFS rates were 68.2%, 45.5 and 0.0%, respectively. The median overall survival was 9.4 months, and the 3-, 6- and 12-month overall survival rates were 75.5%, 55.0 and 13.8%, respectively.

**Conclusions:**

To our knowledge, this is the first study reports outcomes of DEB-TACE with doxorubicin-loaded CallSpheres bead treatment in the management of patients with unresectable or recurrent esophageal carcinoma. According to our results, this is a safe and feasible treatment modality that may be considered among the options for the treatment of these patients.

## Background

Transarterial chemoembolization (TACE) is a palliative treatment for unresectable or recurrent esophageal or gastric carcinoma and its related gastrointestinal bleeding [1,3]. Although conventional TACE can improve the chemotherapy drug concentration by local transarterial infusion [4], drug cannot reside a long period or slowly release [5]. As a novel drug delivery system, drug-eluting beads transarterial chemoembolization (DEB-TACE) can slowly release chemotherapy drugs and thus may improve its safety and efficacy [6, 7]. Nowadays, DEB-TACE has been widely used for the treatment of unresectable hepatocellular carcinoma [8, 9]. Transarterial infusion chemotherapy has been used in patients with T3 esophageal squamous carcinoma after radical surgery[10]. However, the clinical outcomes of DEB-TACE with doxorubicin-loaded CalliSpheres bead for patients with unresectable or recurrent esophageal carcinoma have not been reported. The aim of this study is to assess the clinical feasibility and safety of DEB-TACE for patients with unresectable or recurrent esophageal carcinoma.

## Methods

### Patients

**Fig. 1:**
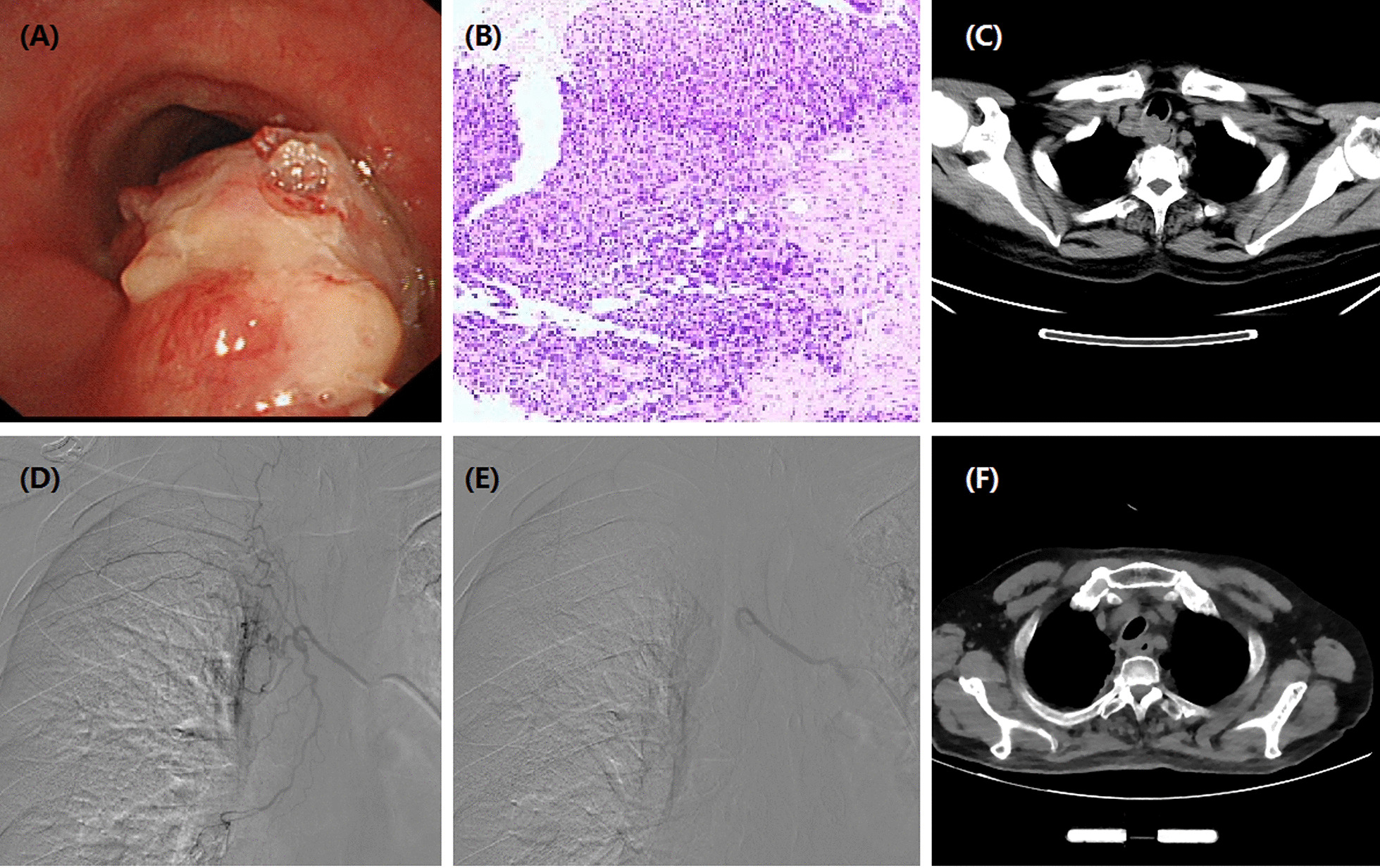
Images of a 66-year-old man with esophageal cancer in upper and middle esophagus. **a**Brancheoscopy shows esophageal tumor ingrowths into tracheal wall. **b**Pathological diagnosis of squamous cell carcinoma. **c**CT scan shows an obvious thickening of esophageal wall and invasion into tracheal wall. **d**The right bronchial artery is super-selectively catheterized by a microcatheter. **e**Tumor-feeding artery was embolized using doxorubicin loading-CalliSpheres beads. **f**After 1 month, CT scan shows a stable mass in the esophageal body

**Fig. 2:**
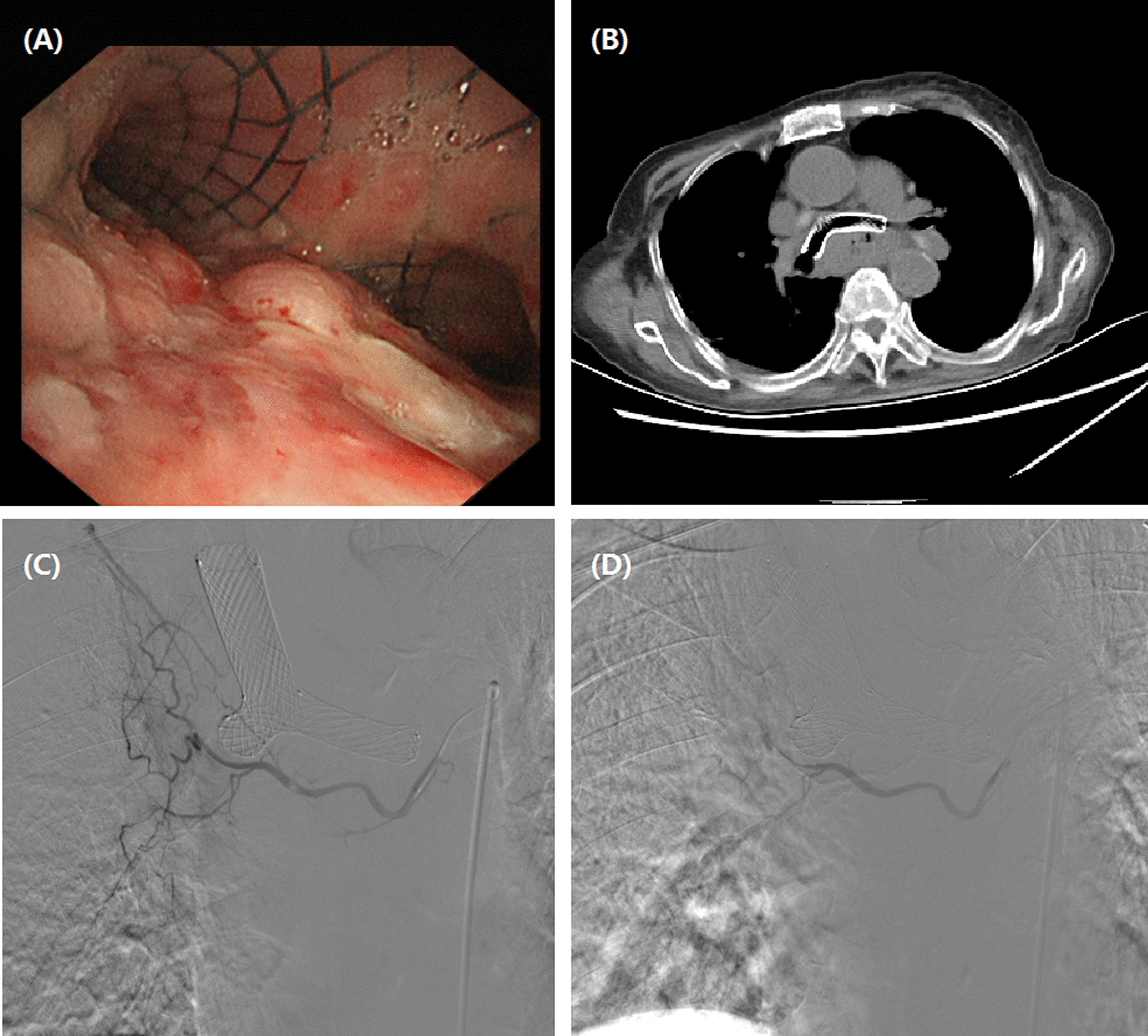
Images of a 75-year-old woman with esophageal cancer in upper and middle esophagus. **a**Brancheoscopy shows esophageal tumor ingrowths into tracheal wall surrounding the airway stent. **b**CT scan shows an obvious thickening of esophageal wall and invasion into tracheal wall surrounding the airway stent. **d**The right bronchial artery is super-selectively catheterized by a microcatheter. **e**Tumor-feeding artery was embolized using doxorubicin loading-CalliSpheres beads

**Fig. 3:**
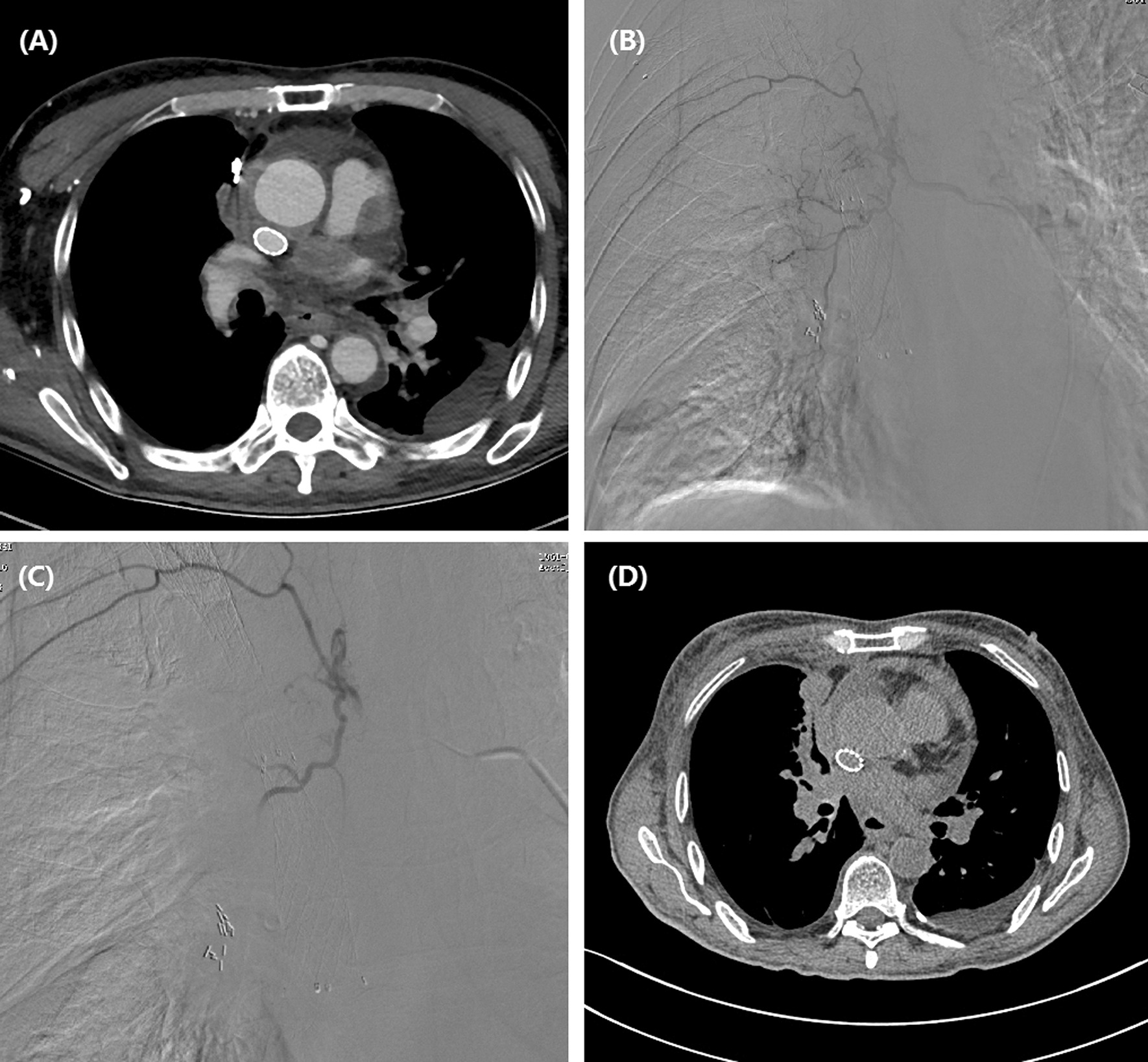
Images of a 58-year-old man with esophageal cancer in upper and middle esophagus. **a**CT scan shows a thickened esophagus with stent in super vein cava. **b**The right bronchial artery is the tumor-feeding artery. **c**The tumor staining disappeared after DEB-TACE with CalliSpheres beads. **d**CT scan shows a stable esophageal mass 1 month after DEB-TACE

**Fig. 4:**
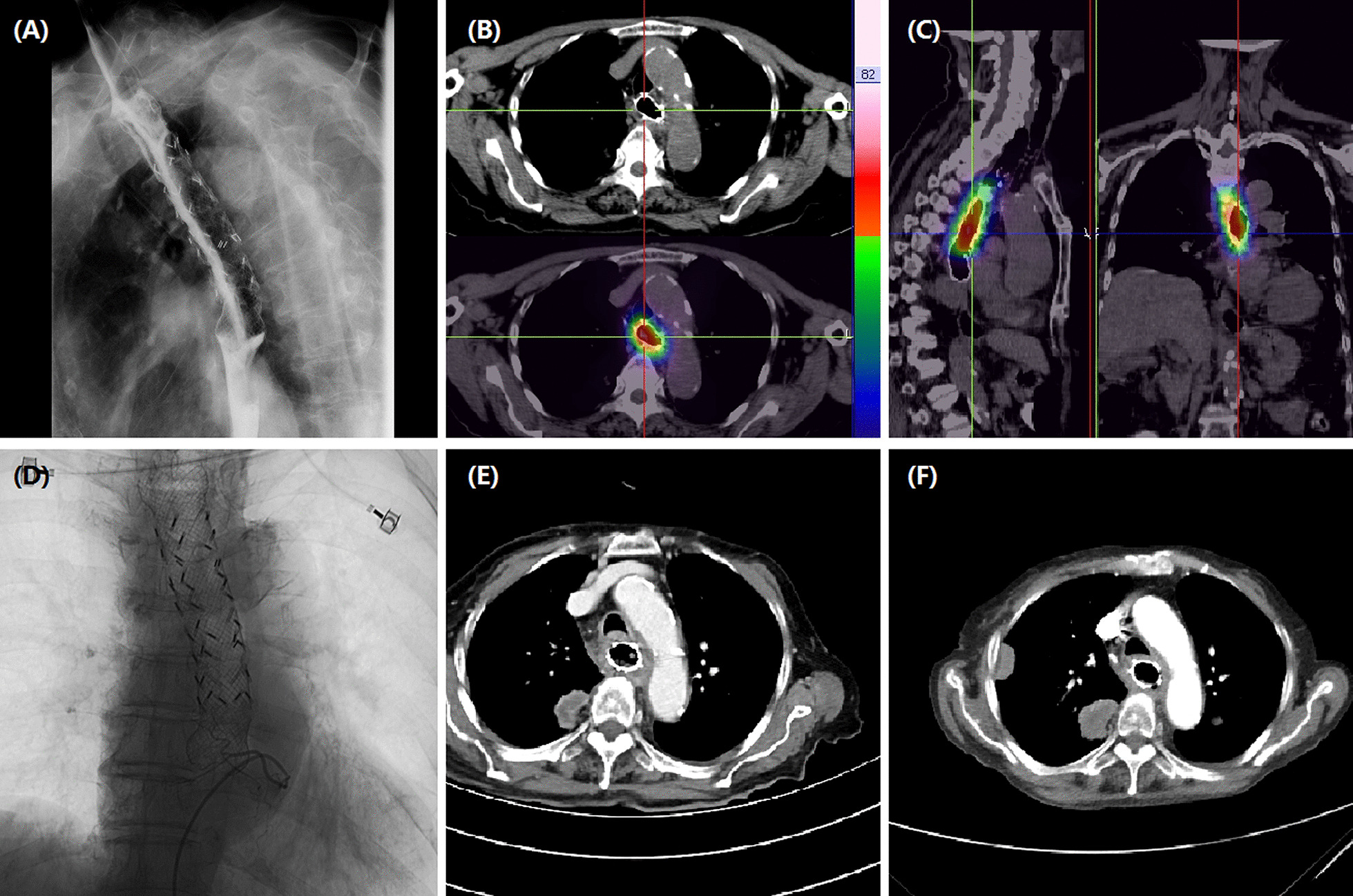
Images of an 83-year-old woman with esophageal cancer in upper and middle esophagus. **a**Radioactive stent was place due to serious stenosis caused by esophageal cancer. **b**Pathological diagnosis of adenocarcinoma. **c**CT scan shows an obvious thickened esophageal wall with a mass in the right upper lung after 6 months. **d**The left gastric artery is super-selectively catheterized and embolized using oxaliplatin loading-CalliSpheres beads. **e**After 9.9 months, CT scan shows a decreased esophageal wall with progressed masses in the right upper lung

This study was approved by the institutional review board of our hospital, and informed consent was waived due to its retrospective nature. This study included 21 consecutive patients with unresectable or recurrent esophageal carcinoma undergoing DEB-TACE between July 2017 and September 2020. Upper gastrointestinal endoscopy with biopsies was performed for pathological diagnosis before DEB-TACE procedure if necessary (Figs.1a,2a). All patients underwent chest computed tomography (CT) scanning to assess the maximal thickness of tumor, tumor location and local/distant metastases before procedure and during follow up (Figs.1c, f, 2b,3a, d,4b, c, e, f). Clinical medical records were retrospectively collected, such as demographic; history of chemotherapy or esophageal surgery; symptoms and signs; course of disease; carcinoma characteristics; comorbidities; laboratory tests.

### Inclusion and exclusion criteria

Indications for DEB-TACE: age older than 18 years; esophageal carcinoma confirmed by pathology (Fig.1b); recurrent esophageal carcinoma after surgery; disease progression after radiochemotherapy; refused or ineligible to radiochemotherapy; no other kinds of life-threatening diseases. Exclusion criteria: combined with other kinds of carcinoma but without standard treatment; white blood cell count is less than 3.010^9^/L; platelets count is less than 40.010^9^/L; serious infection; pregnant woman or breast feeding.

### DEB-TACE procedure

DEB-TACE procedure was conducted under local anesthesia and fluoroscopy. A 5F Cobra catheter (Terumo, Japan) was introduced via the right femoral artery access. Esophageal arteriography was performed to look for tumor staining and its tumor-feeding artery. A 2.4-Fr microcatheter was selectively catheterized into the tumor-feeding artery, and then DEB-TAE was performed (Figs.1d, e, 2c, d,3b, c, 4d). Raltitrexed of 4mg or oxaliplatin of 100mg were transcatheter infused before CalliSpheres beads embolization. Doxorubicin (60mg) was loaded with CalliSpheres beads (300500m, Jiangsu Hengrui Medicine Co. Ltd., Nanjing, China) for about half an hour. Final arteriography was performed to confirm the disappearance of tumor staining.

### Definitions

Technical success was defined as a complete disappearance of tumor staining and its cancer-feeding artery but without any serious complications (such as esophageal perforation, massive bleeding) within 5 days after DEB-TACE procedure. The unresectability was defined according to Esophageal and Esophagogastric Junction Cancers, Version 5.2020, NCCN Clinical Practice Guidelines in Oncology [11]. The local response of the target pulmonary lesions was evaluated by chest CT according to Response Evaluation Criteria in Solid Tumors version 1.1 [12]. The disease control rate (DCR) and objective response rate (ORR) were primary endpoints, which were calculated as a sum of complete response, partial response and stable disease, or a sum of complete response and partial response, respectively. The secondary endpoints were overall survival rate and progression-free survival (PFS).

### Follow-up

All patients underwent chest CT within 2 weeks and every 1 to 1.5 months thereafter. Follow up was finished for all patients with the last date on 6 October 2020.

## Results

### Clinical presentation

This study included 21 patients (15 men; 68.79.7; range 4686 years) with unresectable or recurrent esophageal carcinoma undergoing DEB-TACE between July 2017 and September 2020 (Table1). Eighteen patients (85.7%) were diagnosed with adenocarcinoma. Eight and 3 patients showed local and distant metastases, respectively. Four patients (19.0%) showed recurrence after esophageal surgery, 10 patients (47.6%) received previous chemotherapy, 7 (33.3%) patients also received radiotherapy, and 4 (19.0%) patients had history of esophageal surgery. Symptoms of unresectable or recurrent esophageal carcinoma, such as dysphagia was present in 14 (66.7%), and 2 patients presented with choking cough upon drinking due to esophago-tracheal fistula. The median course of disease from onset of diagnosis to DEB-TACE was 9.0 months (IQR 2.012.0 months).

**Table 1:** Patient and cancer characteristics

Characteristics	Values
Sex, male, n (%)	15 (71.4)
Mean age, Range, years	68.79.7 (4686)
History of chemotherapy, n (%)	10 (47.6)
History of esophageal surgery, n (%)	4 (19.0)
Median course of disease, months	9.0 (2.0, 12.0)
Esophageal carcinoma diagnosis	
Adenocarcinoma/Squamous cell carcinoma	18 (85.7)/3 (14.3)
Local/distant metastases	8 (38.1)/3 (14.3)
Comorbidities, n (%)	
Hypertension	6 (28.6)
Diabetes mellitus	6 (28.6)
Coronary heart disease	1 (4.8)
Symptom and sign, n (%)	
Dysphagia	14 (66.7)
Choking cough	2 (9.5)
Others	5 (23.8)
Location of carcinoma	
Upper middle segment	9 (42.9)
Meddle lower	4 (19.0)
Esophageal cardia junction	7 (33.3)

### DEB-TACE treatments

Twenty-two sessions of DEB-TACE were performed in 21 patients. The technical success rate was 100%. All patients received transarterial infusion with raltitrexed (4mg) or oxaliplatin (100mg). One patient received a second session of DEB-TACE after one month. Twenty-seven tumor feeding arteries were embolized, including the inferior thyroid artery (n=4), the bronchial artery (n=9), the proper esophageal artery (n=5), the intercostals artery (n=3) and the left gastric artery (n=6). One patient received stent placement in superior vena cava due to malignant stenosis. Eight patients (38.1%) received placement of esophageal stent due to serious stenosis (Fig.4a). Six patients (28.6%) underwent airway stenting due to airway stenosis caused by tumor ingrowths. One patient (4.8%) received ^125^I seeds implantation in lung mass. The median inpatient duration was 10.0 days (IQR 9.0, 15.0) and the median cost was 7.110^4^ (IQR 4.8, 8.6) RMB (Table2).

**Table 2:** Clinical outcomes of DEB-TACE with CalliSpheres beads

Variables	Value
Median operation time, min	70.0 (52.5, 101.0)
Median inpatient duration, days	10.0 (9.0, 15.0)
Median cost, IQR, 10^4^ RMB	7.1 (4.88.6)
The embolized arteries	
The inferior thyroid artery	4 (19.0)
The bronchial artery	9 (42.9)
The proper esophageal artery	5 (23.8)
The intercostal artery	3 (14.3)
The left gastric artery	6 (28.6)
Complications, n (%)	
Chest pain	3 (14.3)
Nausea or vomiting	2 (9.5)
Other treatments, n (%)	
Superior vena cava stenting	1 (4.8)
Esophagus stenting	8 (38.1)
Airway stenting	6 (28.6)
^125^I seeds implantation	1 (4.8)

### Endpoint

The median follow-up time was 3.6 months (IQR 1.59.4 months). ORR and DCR were 42.9 and 85.7%, 28.6 and 71.4%, 20.0 and 40.0% respectively at 1-, 3-, and 6-months after DEB-TACE (Table3). The median PFS was 6.0 months, and the 3-, 6- and 12-month PFS rates were 68.2%, 45.5 and 0.0%, respectively. The median overall survival was 9.4 months, and the 3-, 6- and 12-month overall survival rates were 75.5%, 55.0 and 13.8%, respectively.

**Table 3:** Local tumor response

Response, n (%)	1 month	3 months	6 months
Complete response	0 (0.0)	0 (0.0)	0 (0.0)
Partial response	9 (42.9)	4 (28.6)	2 (20.0)
Stable disease	9 (42.9)	6 (42.9)	2 (20.0)
Progressive disease	3 (14.3)	4 (28.6)	6 (60.0)
ORR	9 (42.9)	4 (28.6)	2 (20.0)
DCR	18 (85.7)	10 (71.4)	4 (40.0)

*DCR*disease control rate, *ORR*objective response rate

### Complications and safety

No serious complications were observed, including procedure-related deaths, massive bleeding, and esophageal perforation. The post-embolisation syndrome such as nausea or vomiting was found in 2 patients. Three patients (14.3%) complained of minor chest pain.

## Discussion

Open surgery is the preferred for potentially resectable esophageal carcinoma. TACE may serve as palliative treatment for unresectable or recurrent esophageal carcinoma. The left gastric artery was the main tumor-feeding arteries for cancer in esophageal cardia junction [2], and the bronchial artery may responsible for the blood supply for upper and middle esophageal carcinoma. Transarterial embolization can be used for in esophageal cancer patients with esophageal bleeding [13, 14]. Transarterial infusion chemotherapy has been used in patients with T3 esophageal squamous carcinoma after radical surgery [10]. As far as we know, due to the concern of esophageal perforation complications, TACE treatment of esophageal cancer and other cavity organs is rarely reported, not to mention the use of DEB treatment of esophageal cancer. So far, the clinical outcomes of DEB-TACE with doxorubicin-loaded CalliSpheres bead for patients with unresectable or recurrent esophageal carcinoma have not been reported.

The choice of optimal embolic materials is a major concern for hollow viscera. Unfortunately, there is no guideline in the choice of embolic materials. Temporary embolic material, absorbable gelatin sponge, was the most commonly used in order to avoid visceral perforation. However, permanent embolic material including PVA and N-butyl cyanoacrylate can be used for esophageal bleeding in esophageal cancer patients to avoid artery recanalization [13, 14]. As a novel drug delivery system, DEB-TACE has been widely used for the treatment of unresectable hepatocellular carcinoma [8, 9], refractory lung cancer and so on [15,19]. However, the safety and efficacy of DEB-TACE has not been assessed in patients with unresectable or recurrent esophageal carcinoma. In our study, the technical success rate of DEB-TAE was 100% for patients with unresectable or recurrent esophageal cancer. The esophagus is easily damaged by ischemia. In this study, no serious complications were observed, including procedure-related deaths, massive bleeding, and esophageal perforation.

DEB-TACE can embolize the tumor-feeding artery to block the blood supply [6, 7], and controlled release of chemotherapy drugs, thus may improve the clinical outcomes [20,22]. Our data showed that DEB-TACE appears to be well-tolerated for treating patients with unresectable or recurrent esophageal carcinoma. The technical success rate was 100%, however, DEB-TACE was performed in only 21 patients and the sample size of our study was small. The catheterization of the tumor-feeding arteries was the most common pitfall, considering that tumor-feeding arteries of esophageal cancer are complex and changeable. The left gastric artery was the main tumor-feeding arteries for cancer in esophageal cardia junction [2], and the bronchial artery may responsible for the blood supply for upper and middle esophageal carcinoma. Besides, potential complication of esophageal perforation after DEB-TACE for cavity organ is of great concern to researchers. In this study, no esophageal perforation was observed after DEB-TACE.

The ORR and DCR were 42.9 and 85.7%, 28.6 and 71.4%, 20.0 and 40.0% respectively at 1-, 3-, and 6-months after DEB-TACE. Our results indicated that DEB-TACE showed a moderate disease control rate during a short-term follow up. It is not certain that DEB-TACE benefits the survival for patients with advanced hepatocellular carcinoma when compared with conventional TACE [23, 24]. In this study, the 3-, 6- and 12-month PFS rates were 68.2%, 45.5 and 0.0%, respectively. The 3-, 6- and 12-month overall survival rates were 70.0%, 51.0 and 12.8%, respectively. Thus, comparative studies with TACE, chemotherapy, radiotherapy and so on are needed to verify whether this treatment has survival benefit.

Several limitations exist in the current study. This retrospective study was performed in a single center and patient sample size was relatively small; there was a possible selection bias. Lack of comparison with chemotherapy, TACE or radiotherapy is a major shortcoming, and comparison is also wanted to evaluating the true efficacy of DEB-TACE.

## Conclusions

To our knowledge, this is the first such study in the literature. According to our results, DEB-TACE is a safe and feasible treatment alternative that may be considered among the options for the treatment of these patients. Comparative studies are warranted to further analyze its clinical efficacy.

## Data Availability

For further details, the corresponding author can be contacted.

## Abbreviations

CT: Computerized tomography
DEB-TACE: Doxorubicin-eluting CalliSpheres bead-transarterial chemoembolization
DCR: Disease control rate
ORR: Objective response rate
PFS: Progression-free survival
IQR: Interquartile range
